# Lockdown: The Impact on Management of Glaucoma in a Suburban Tertiary Centre in Malaysia

**DOI:** 10.7759/cureus.34412

**Published:** 2023-01-30

**Authors:** Jasvinjeet K Sidhu, Azhany Yaakub, Liza Sharmini Ahmad Tajudin

**Affiliations:** 1 Department of Ophthalmology and Visual Science, School of Medical Sciences, Universiti Sains Malaysia, Kubang Kerian, MYS; 2 Ophthalmology Clinic, Hospital Universiti Sains Malaysia, Kubang Kerian, MYS

**Keywords:** management, lockdown, post-movement control order, pre-movement control order, glaucoma

## Abstract

Introduction: The COVID-19 pandemic brought the world to a standstill in 2020. Many countries have imposed lockdowns, known as the movement-control order (MCO) in Malaysia, to prevent transmission of the disease.

Aim: The objective of this study is to evaluate the impact of the MCO on the management of glaucoma patients in a suburban tertiary hospital.

Methodology: We conducted a cross-sectional study of 194 glaucoma patients between June 2020 and August 2020 in the glaucoma clinic at Hospital Universiti Sains Malaysia. We evaluated the patients’ treatment, visual acuity, intraocular pressure (IOP) measurements, and potential signs of progression. We compared the results with those of their last clinic visits prior to the MCO.

Results: We studied 94 (48.5%) male and 100 (51.5%) female glaucoma patients with a mean age of 65 ± 13.7. The mean duration between pre-MCO and post-MCO follow-up was 26.4 ± 6.7 weeks. There was a significant increase in the number of patients with deterioration of visual acuity, and one patient lost his vision after the MCO. There was also a significant elevation of the mean IOP of the right eye: pre-MCO, 16.7 ± 7.8 mmHg, compared to post-MCO, 17.7 ± 8.8 mmHg (*p* = 0.027). The cup-to-disc ratio (CDR) for the right eye increased significantly from pre-MCO, 0.72 ± 0.18, to 0.74 ± 0.19 post-MCO (*p* < 0.001). However, there were no significant changes in IOP or CDR in the left eye. Twenty-four patients (12.4%) missed medications during the MCO period, and 35 patients (18%) required additional topical medications due to the progression of the disease. Only one patient (0.5%) required admission due to uncontrolled IOP.

Conclusion: Lockdown, as a preventive step in the COVID-19 pandemic, indirectly caused the progression of glaucoma and uncontrolled IOP.

## Introduction

Coronavirus is an enveloped, single-strand RNA virus that belongs to the Orthocoronavirinae subfamily [1]. Coronavirus is known to cause various clinical conditions, including the latest COVID-19 pandemic [2]. COVID-19 is extremely infectious and is well-known to be easily transmitted [3]. The World Health Organization (WHO) declared the COVID-19 outbreak a pandemic on March 11, 2020. To date, COVID-19 has affected 651 million people and is responsible for 6.65 million deaths. It brought the world to a standstill in 2020 [4].

In Malaysia, the first case was reported on January 25, 2020, followed by a surge in cases from March 2020 onwards [5]. Similarly to other countries in the world, Malaysia imposed a lockdown, better known as a movement-control order (MCO), to prevent transmission of the disease [6]. The MCO was imposed on March 18, 2020, with interstate travel restrictions, including the limitation of the number of people in a car and on public transportation [5]. Schools, government offices, and private premises were closed except for essential service providers [5,7]. Dining in restaurants was not allowed, and staying at home was encouraged [6].

As a step to conserve resources for the battle against this pandemic, public hospitals in Malaysia implemented changes at many levels [8]. Priority was given to treating and performing contact tracing in COVID-19 cases [9]. Only emergency cases were given priority [10-11]. Clinic follow-up appointments for ‘stable’ chronic illnesses were rescheduled, and elective surgeries were cancelled [8]. Most of the ophthalmic cases were considered non-urgent and were postponed to a later date [12]. At the same time, patients were also anxious and developed an unnecessary fear of contracting the infection if they visited the hospital [13]. There was a 70% reduction in scheduled clinic visits in six hospitals in Italy during the lockdown [14].

Glaucoma is a chronic eye disease, similar to diabetes mellitus, and it requires lifetime treatment and follow-up. In general, glaucoma is asymptomatic, even in advanced cases [15]. Symptoms are usually related to the sudden increase in intraocular pressure (IOP) [16]. Patients with pre-existing glaucoma are known to have a tolerance for high IOP [15]. The MCO has hampered the routine follow-up for patients with glaucoma [17]. Routine follow-up in glaucoma patients is necessary to monitor IOP and detect progression [18].

Our aim in this study was to evaluate the impact of lockdown on the clinical management of patients with glaucoma in a suburban tertiary centre in Malaysia.

## Materials and methods

We conducted a cross-sectional study between June 2020 and August 2020 involving patients attending the glaucoma clinic of Hospital Universiti Sains Malaysia (HUSM). HUSM is one of the tertiary eye care centres in the state of Kelantan, Malaysia. Kelantan is situated in the northeast of Malaysia, with an estimated population of 1.95 million [19].

During the three-month period from June through August 2020, we recruited all patients with glaucoma who attended our glaucoma clinic. Our inclusion criteria were confirmed cases of glaucoma with at least three previous visits within a year prior to March 18, 2020, and a pre-MCO IOP and best-corrected visual acuity (BCVA) recorded. We excluded those who were diagnosed with ocular hypertension, primary angle closure (PAC), or primary angle closure suspects (PACS). We also excluded those with pre-existing optic neuropathies.

We included 221 patients in the preliminary recruitment phase, and we traced their medical records. We excluded 27 patients from the final recruitment due to an inadequate number of pre-MCO follow-ups (19 patients) or because the IOP and BCVA were taken more than four months prior to MCO (eight patients). We obtained the pre-MCO IOP and BCVA from the medical record.

A thorough ocular examination was conducted, including slit-lamp biomicroscopy examination, fundus examination, and IOP measurement. Trained staff nurses did a visual acuity assessment using the Snellen chart. BCVA was included in our analysis. We used Goldmann applanation tonometry (Haag-Streit, Switzerland) to measure the IOP. A fundus examination was conducted using a 90D lens (VOLK, USA). Two experienced glaucoma specialists performed the vertical cup-to-disc ratio (VCDR) assessment. We recorded four clinical outcomes, which include missed medication, change of treatment, hospital admissions, or no change in treatment.

Missed medication was recorded when the patient was out of medication for more than two weeks prior to the recruitment period. A change of treatment was defined as any addition, switching, or changing of topical pressure-lowering drugs for uncontrolled IOP or failure to reach target pressure. Admission for uncontrolled IOP refers to any admission to the hospital due to uncontrolled IOP during the recruitment period. No change in treatment was when the target pressure was achieved without missing medication.

Primary open-angle glaucoma is a chronic progressive optic neuropathy with characteristic morphological changes at the optic nerve head and retinal nerve fibre layer, in the absence of other ocular disease or congenital anomalies. Angle-closure glaucoma is defined by the presence of iridotrabecular contact. A secondary glaucoma is a heterogeneous group of conditions, in which elevated IOP is the leading pathological factor causing glaucomatous optic neuropathy, either being open or closed angle [20]. We defined pre-MCO IOP and BCVA as the last IOP and BCVA taken no longer than four months prior to the MCO.

We performed our statistical analysis with the Statistical Analysis Software Package (SPSS), version 26 (SPSS Inc., Chicago, IL). We conducted double entries of the data to avoid incorrect entries or missing data. We analysed categorical data using the Pearson chi-squared test. We analysed numerical data such as IOP and VCDR using a paired t-test. A p-value of less than 0.05 was considered statistically significant.

## Results

A total of 194 patients (345 eyes) were enrolled in this study. The mean duration from the last visit (prior to MCO) and the recruitment was 26.4 ± 6.7 weeks. There was almost an equal distribution between male and female glaucoma patients (Table 1). The majority of patients were Malays: 160 (82.5%), Chinese: 33 (17.0%), and Indian: 1 (0.5%). This generally reflected the population distribution in Kelantan. The majority of the patients with glaucoma had bilateral involvement (77.8%) and mostly primary glaucoma (85.8%) (Table 1).

**Table 1:** Demographic characteristics of glaucoma patients

Characteristics	Number (n)	Percentage (%)
Gender		
Male	94	48.5
Female	100	51.5
Ethnicity		
Malay	160	82.5
Chinese	33	17.0
Indian	1	0.5
Laterality		
Bilateral	151	77.8
Unilateral right eye	20	10.3
Unilateral left eye	23	11.9
Type of glaucoma		
Right eye primary glaucoma	146	42.3
Right eye secondary glaucoma	25	7.2
Left eye primary glaucoma	150	43.5
Left eye secondary glaucoma	24	7.0

Pre- and post-MCO visual acuity of both eyes are shown in Table 2. Although not statistically significant, the table shows noticeable visual acuity deterioration during the MCO period. There was one patient who had good vision pre-MCO but lost his vision in the right eye during this period.

**Table 2:** Comparison of BCVA between pre- and post-movement-control order Pearson chi-square test, p < 0.05 is considered significant. NPL: no perception to light, BCVA: best corrected visual acuity.

Right eye			Visual acuity	Left eye		
Pre-MCO n (%)	Post-MCO n (%)	p-value		Pre-MCO n (%)	Post-MCO n (%)	p-value
123 (72.4)	122 (71.8)	p > 0.950	6/6-6/12	129 (74.1)	125 (71.8)	p > 0.950
10 (5.9)	10 (5.9)	p > 0.950	6/18-6/36	13 (7.5)	16 (9.2)	p > 0.950
16 (9.4)	16 (9.4)	p > 0.950	6/60 and worse	17(9.8)	18(10.3)	p > 0.950
21 (12.4)	22 (12.9)	p > 0.950	NPL	15 (8.6)	15 (8.6)	p > 0.950

**Figure 1:**
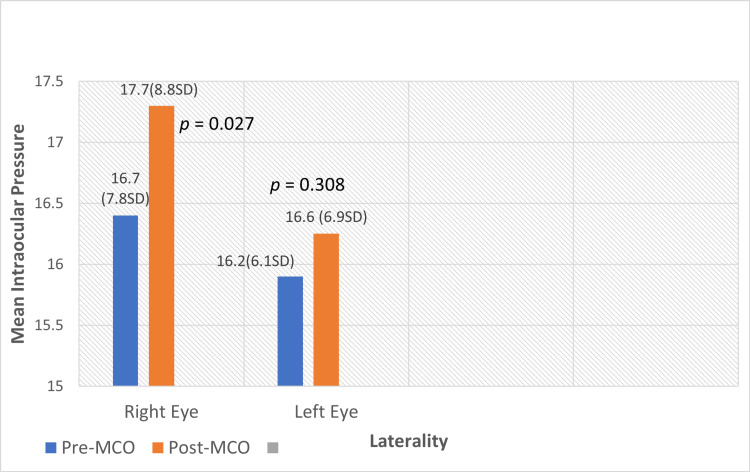
Comparison between mean IOP pre- and post-MCO Paired sample t-test on pre- and post-MCO mean intraocular pressure. p < 0.05 is considered significant. MCO: movement-control order, IOP: intraocular pressure.

The mean IOP pre- and post-MCO also showed a difference, with worsening IOP noted after the MCO period, as shown in Figure 1. There is a statistically significant difference noted in the IOP of the right eye (p < 0.05) using the paired t-test.

**Figure 2:**
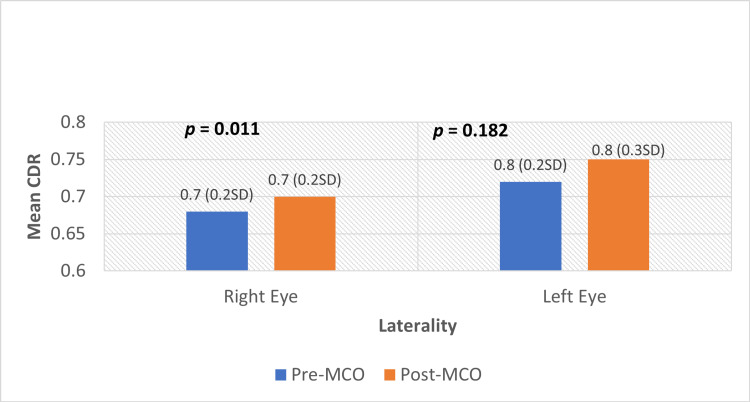
Comparison between mean cup-to-disc ratio pre- and post-MCO Paired sample t-test on pre- and post-MCO mean CDR. p < 0.05 is considered significant. MCO: movement-control order, CDR: cup-to-disc ratio.

Similarly, there is a statistically significant increase in the cup-to-disc ratio of the right eye, as demonstrated in Figure 2.

**Figure 3:**
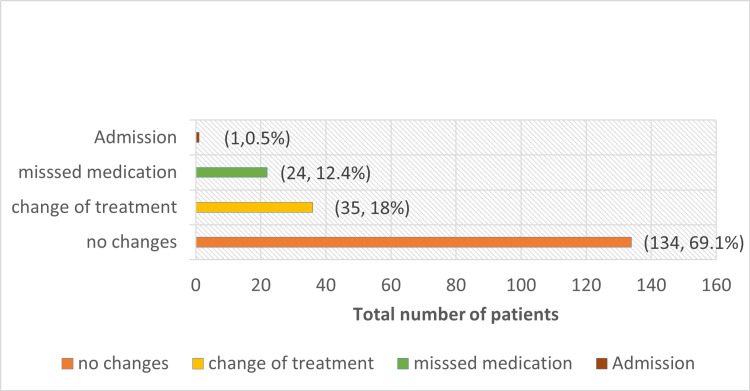
Changes of management in glaucoma patients post-MCO MCO: movement-control order.

Figure 3 demonstrates changes in the management of glaucoma patients during the MCO period. It shows that although the majority of patients had no changes in management, a significant number of patients missed their medication during this period, 24 (12.4%). Thirty-five patients (18%) required a change in their current treatment due to the progression of glaucoma observed post-MCO. One patient required admission due to significantly high intraocular pressure, requiring monitoring in the ward. However, none of our patients required surgery during this period.

## Discussion

The COVID-19 pandemic has caused major disruptions in all aspects of life worldwide. Conjunctivitis and keratoconjunctivitis were reported as the clinical manifestations of this easily transmitted virus [21]. However, the indirect effect of chronic eye diseases such as glaucoma, age-related macular degeneration (AMD), and diabetic retinopathy has not been appreciated. Chronic eye diseases require lifelong follow-up and are known to cause irreversible blindness [22,23]. The lockdown in Malaysia has no doubt caused disruption in the management of chronic eye diseases.

Based on the National Eye Survey II, glaucoma is the second cause of visual impairment and blindness in Kelantan [24]. Kelantan borders Terengganu, Pahang, and Thailand. Our centre receives referrals from southern Thailand, Terengganu, and various parts of Malaysia. With the MCO, many of our patients experienced disruptions in their medication supply and follow-up. The mean duration of follow-up was increased to 26.4 (SD 6.7) weeks. In general, the follow-up visit was 16 to 24 weeks for patients with stable glaucoma. Similar findings were reported in Italy, the United Kingdom, and the United States [25-27]. These countries recorded higher prevalence and morbidity due to COVID-19 compared to Malaysia [28-30]. Fifty-nine patients (77 eyes) reported changes in treatment, including missing medications for more than two weeks. Sadly, one eye was blind due to the indirect effect of the lockdown.

COVID-19 affected the healthcare fraternity worldwide with regard not only to the disease but also to non-COVID-related illnesses. Our study demonstrates that the pandemic, which resulted in the government imposing the MCO, has indirectly affected the glaucoma patients in our centre. Although our study was conducted in an ophthalmology department in one healthcare facility in the country, it could reflect the situation in the entire country.

There are some limitations to this study. It involved a single centre with a relatively small number of patients. A more objective assessment of disease progression, like Humphrey's visual field and optical coherence tomography of the optic nerve and the retinal nerve fibre layer, could have been performed to further justify the results of our study. Also, glaucoma can continue to progress with time, even during the non-MCO period. In this study, we are unable to compare glaucoma progression between MCO and non-MCO periods. In the future, another similar study design can be done post-COVID-19 pandemic, and results compared with this study.

## Conclusions

The COVID-19 pandemic caught the world unprepared and severely crippled healthcare systems globally. This study showed that the movement control order implemented to halt the spread of COVID-19 has impacted the routine follow-up of glaucoma patients. A significant number of patients missed their medications and required changes in medications due to the progression of the disease during this period.
